# Rectal budesonide: A potential game changer after Kasai hepatoportoenterostomy

**DOI:** 10.1002/jpn3.70147

**Published:** 2025-07-02

**Authors:** Stefanie Langreen, Nathalie Pauer, Eva D. Pfister, Norman Junge, Ulrich Baumann, Omid Madadi‐Sanjani, Claus Petersen, Jens Dingemann, Nagoud Schukfeh

**Affiliations:** ^1^ Department of Pediatric and Adolescent Surgery Hannover Medical School Hannover Germany; ^2^ Department of Kidney, Liver and Metabolic Disease, Division of Pediatric Gastroenterology and Hepatology Hannover Medical School Hannover Germany; ^3^ Institute of Immunology and Immunotherapy University of Birmingham Birmingham UK

**Keywords:** biliary atresia, corticoids, liver surgery, liver transplantation, pediatric

## Abstract

**Objectives:**

Intravenous or oral steroid administration in patients with biliary atresia (BA) after Kasai hepatoportoenterostomy (HPE) is commonly practiced, however, the benefits remain controversial. Some studies suggest no positive effects while risking steroid associated side effects. Rectal application of glucocorticoids has so far only been assessed by our group with promising short‐term results. We now aim to evaluate the impact of rectal budesonide administration on long‐term native liver survival in patients undergoing HPE for BA.

**Methods:**

We performed a retrospective cohort study and included all patients after HPE who received rectal budesonide (2 mg) for 3 months from 2011 to 2022, compared to a historical control group without any glucocorticoid treatment. Jaundice‐free native liver survival (jfNLS) was assessed at 6 months, 2 years, 5 years, and 10 years post‐Kasai. Serious adverse effects of budesonide were documented.

**Results:**

Our analysis confirmed our previously published improvements in jfNLS at 6 months (53% vs. 39%) and 2 years (45% vs. 22%), while revealing sustained benefits at 5 years (40% vs. 23%) and 10 years (32% vs. 13%). However, these benefits were exclusive to patients with nonsyndromic BA. No serious budesonide‐associated adverse side effects were found.

**Conclusions:**

Our findings support the efficacy of rectal budesonide application in enhancing long‐term outcomes, providing a safe therapeutic approach and improving jfNLS after HPE for BA, without severe adverse effects. Prospective randomized controlled trials are required to further evaluate its potential post‐Kasai benefits and compare it to systemic glucocorticoid therapy.

## Abstract (highlights)

**What is Known:**

- Intravenous and oral steroids are commonly administered after Kasai hepatoportoenterostomy.
- Benefits are contentious.

**What is New:**

- Budesonide application improves jaundice‐free native liver survival after 5 and 10 years.
- Rectal budesonide causes no serious corticosteroid‐associated, adverse effects.

## 1 INTRODUCTION

Biliary atresia (BA) is a rare congenital condition, defined by the obliteration or discontinuity of the biliary system, leading to cholestasis, cirrhosis, and finally liver failure.^1^, ^2^, ^3^ It remains the leading indication for transplantation in the pediatric population.^4^ Without intervention, the condition is fatal due to end‐stage liver disease.^5^

The Kasai hepatoportoenterostomy (HPE) restores bile flow for a yet undefined period, thereby delaying or completely avoiding liver transplantation. However, the long‐term survival with the native liver remains uncertain, with failure rates at 5 years generally ranging around 50% even at the highest level of care.^6^, ^7^ This may even be significantly worse in other regions of the world.^8^, ^9^

Success of HPE influencing factors include age and degree of liver fibrosis at time of surgery, whilst early jaundice clearance was linked as favorable prognostic factor: Shneider et al. have demonstrated that a total bilirubin at less than 2 mg/dL (=34.2 μmol/L) by 3 months post‐HPE is associated with improved native liver survival (NLS) at 2 years (86%) compared to those with bilirubin levels ≥ 2 mg/dL (20%).^9^, ^10^, ^11^, ^12^, ^13^ To improve NLS, various adjuvant therapies have been explored, among these, glucocorticoid administration has been the most extensively studied.^14^, ^15^, ^16^ Currently, Ileal bile acid reuptake (IBAT) inhibition are under investigation to reduce toxic bile acid accumulation, as yet with limited success (NCT04524390) or with pending results (NCT04336722). Inflammation appears to be driving pathology making novel and focussed application of steroids attractive. However, the evidence on the efficacy shows conflicting results, debating benefits of steroid application in relation to adverse effects.^14^, ^17^, ^18^

Our study group has observed promising outcomes with the administration of rectal budesonide in patients after HPE, employing the first pass effect to specifically target the liver, leading to a significant improvement in our 2‐year NLS, while avoiding most glucocorticoid‐associated side effects.^19^ Therefore, we conducted a further analysis of the long‐term follow‐up of these patients to confirm a sustained benefit of early rectal budesonide treatment. Furthermore, we aimed to reassess the 6‐month and 2‐year NLS of the patients receiving HPE to validate our previous findings.

## 2 METHODS

### 2.1 Ethics statement

Ethical approval (No. 9429_BO_K_2020) was obtained from the ethical committee of Hannover Medical School. Parental consent was obtained.

### 2.2 Patient collective

All patients who received HPE until December 2022 with subsequent rectal budesonide administration were included in this analysis. From May 2011 onwards, rectal budesonide was offered as an off‐label therapy to all patients following HPE, in cases of budesonide decline, patients were included in the control group. The control group was comprised of all patients receiving HPE from the year 2000 on, without having received any form of glucocorticoid treatment. In this analysis, we compared the long‐term effects of rectal budesonide at 5 and 10 years post‐Kasai compared to our historical control group. Furthermore, we have reevaluated the 6 months and 2 years effect of rectal budesonide by adding another 40 patients that have completed 2 year follow‐up after budesonide since our last publication.^19^

### 2.3 Diagnostics and treatment

Patients with neonatal cholestasis receive a wide range of standardized diagnostics in our tertiary care center, if BA is suspected, confirmation of diagnostics is obtained by open cholangiography. In cases of confirmed BA, we proceed with open HPE and our standardized postoperative treatment (with i.v. antibiotics for 10–14 days, oral antibiotics for 6 months and long term fat soluble vitamins and ursodeoxycholic acid) as previously published.^19^

Until 2021, rectal foam budesonide (Budenofalk™, 2 mg/dose) was administered from Day 5 as off‐label therapy once a day, following the obtaining of parental consent. From 2021 onwards, the start of treatment was moved to Day 3 after HPE. Treatment continues daily for the next 3 months.

Standardized follow‐up comprised of regular visits in our gastroenterological in‐house‐clinic every 3 months, eventually increasing the intervals to 6 months and finally 12 months for patients with uneventful course. Some patients preferred follow‐ups close to home. All the involved health care professionals received a detailed list about ongoing medication and treatment duration, including the budesonide treatment.

### 2.4 Data collection

We retrospectively (prospectively from 2021) collected data on NLS in general, as well as jaundice free NLS (jfNLS)—defined as the absence of scleral or skin jaundice, liver transplantation and death at 6 months, 2 years, 5 and 10 years after HPE was assessed. Additionally, age at HPE, gender, serum transaminase levels, grade of fibrosis according to ISHAK score^20^ and syndromic form of BA with associated malformations was analyzed. Every patient file was screened for following side effects: Growth restriction, Cushing's syndrome, serum glucose fluctuations, infections treated with antibiotics, and osteopenia.

### 2.5 Statistical analysis

Statistical analysis was performed with Microsoft Excel, GraphPad Prism and the open access software R. Quantitative data are represented as mean ± SD in normally distributed samples and assessed by students *t*‐test. In nonnormally distributed samples, data are presented as median with interquartile range and assessed by Mann–Whitney‐*U* test. To preserve data integrity, all outliers were retained in the analysis. Patients who were lost to follow‐up at a specific time were excluded from the analysis for that time only. No imputation was performed for missing data. Qualitative data was assessed with chi‐square test or Fisher's exact test. Survival rates were demonstrated using Kaplan–Meier‐curve and log‐rank test. A confidence interval was set at 95% with *p* < 0.05 deemed significant.

## 3 RESULTS

We included 279 patients, 142 patients in the study group with rectal budesonide and 137 in the control group without glucocorticoid administration. Eighteen patients had refused consent for budesonide and were therefore included in the control group. 6 months and 2 year follow‐up was completed in 249 patients (96%), the 5‐year follow‐up was completed in 194 patients (95%) and in the 10‐year follow‐up in 142 patients (94%).

### 3.1 Demographic and preoperative data

No significant difference regarding age at surgery, sex, or proportion of syndromic versus nonsyndromic BA could be detected between the two groups.

A significant difference in bilirubin levels in favor of the study group could be detected. Unlike in aspartate transferase, alanine transferase, or gamma‐glutamyl transferase levels, in which no significant difference was found (Table 1).

**Table 1:** Patient demographics and preoperative data.

	Study group (*n* = 142)	Control group (*n* = 118)	*p* value
Age at surgery (days)	57.9 ± 21.2	62.4 ± 28.2	n.s.
Sex (male)	57 (40%)	56 (47%)	n.s.
Syndromic BA	11% (*n* = 16)	13% (*n* = 15)	n.s.
Serum bilirubin (μmol/L)	132.5 (109‐153)	147 (118‐186)	**<0.05**
Serum AST (U/L)	235.5 ± 176.6	222 ± 171.7	n.s.
Serum ALT (U/L)	158.8 ± 115.8	146.5 ± 120.7	n.s.
Serum gGT (U/L)	555.8 ± 351.1	512 ± 307	n.s.

*Note*: Metric data presented as mean and standard deviation. Bold values indicate statistical significance.

Abbreviations: ALT, alanine amino transferase; AST, aspartate amino transferase; BA, biliary atresia; gGT, gamma‐glutamyl transferase; n.s., not significant.

No significant difference regarding level of fibrosis according to ISHAK scoring system could be detected (Table SX).

### 3.2 Postoperative bilirubin levels

**Figure 1:**
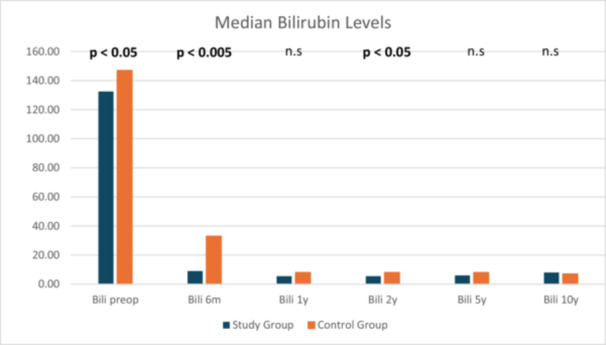
Bilirubin (Bili) levels of the study group compared to the control group preoperatively (preop) and postoperatively. Data presented as median value. m, months; n.s, not significant; y, years.

A significant difference could be detected at 6 months and at 2 years postoperatively between the study and the control group that did not persist over the years (Figure 1 and Table SY).

### 3.3 Postoperative survival

jfNLS was significantly higher in the overall study group at all times. Analysis of the subgroup of nonsyndromic BA patients confirmed a significant difference in jfNLS in favor of the non‐syndromic patients of study group. However, No significant difference in syndromic BA patients between study and control group was found regarding jfNLS (Table 2).

**Table 2:** Postoperative jfNLS in the entire cohort, in nonsyndromic BA patients, and in syndromic BA patients.

	**Study group overall**	**Control group overall**	***p* value**
6 months jfNLS	53% (73/135)	39% (44/112)	**<0.05**
2 years jfNLS	45% (62/135)	28% (31/112)	**<0.005**
5 years jfNLS	40% (35/87)	23% (25/107)	**<0.05**
10 years jfNLS	32% (14/44)	13% (13/98)	**<0.01**
	**Study group nonsyndromic**	**Control group nonsyndromic**	***p* value**
6 months jfNLS	54.5% (66/121)	40.6% (41/101)	**<0.05**
2 years jfNLS	48.8% (59/121)	28.7% % (29/101)	**<0.005**
5 years jfNLS	42.3% (33/78)	23.3% % (23/96)	**<0.05**
10 years jfNLS	32.6% (14/43)	12.5% (11/88)	**<0.01**
	**Study group syndromic**	**Control group syndromic**	***p* value**
6 months jfNLS	42.8% (6/14)	27.2% % (3/11)	n.s.
2 years jfNLS	21.4% (3/14)	18.2% % (2/11)	n.s.
5 years jfNLS	22% (2/9)	18% (2/11)	n.s.
10 years jfNLS	0% (0/1)	10% (1/10)	n.s.

*Note*: Date presented as percentage with jfNLS. Bold values indicate statistical significance.

Abbreviations: BA, biliary atresia; jfNLS, Jaundice‐free native liver survival; n.s., not significant.

**Figure 2:**
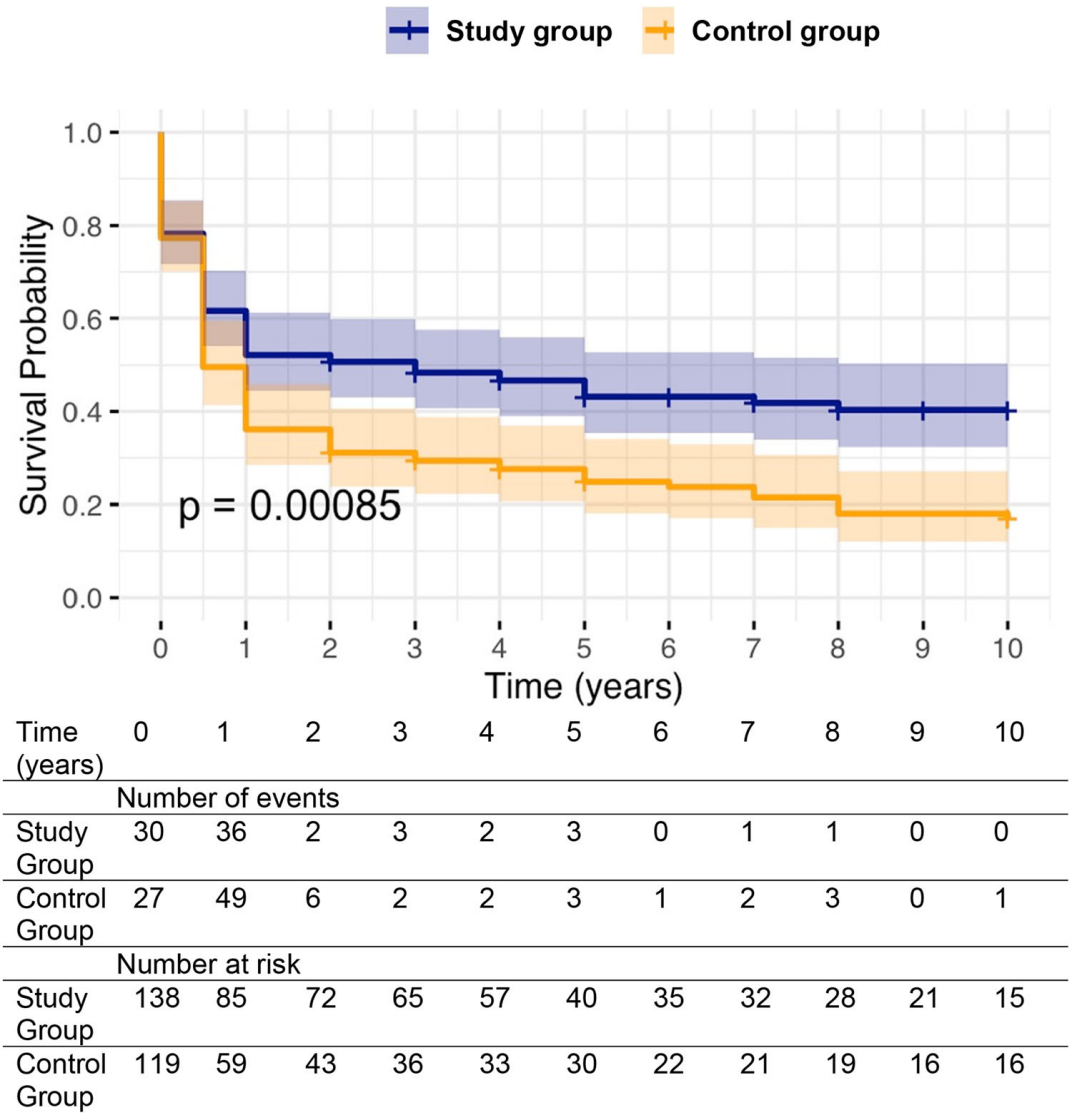
Kaplan–Meier curve comparing native liver survival between the study and control groups over a 10‐year follow‐up. The number of patients at risk and the cumulative number of events are presented below the time axis at each time point. Study group—blue. Control group—orange.

The Kaplan–Meier survival curve demonstrated a significant long‐term survival advantage of the study group (overall NLS, *p* < 0.001) (Figure 2).

### 3.4 Postoperative survival in relation to ISHAK score

No significant difference in NLS was detected between ISHAK ≤ IV and ISHAK ≥ V (*p* = 0.4) (Table SZ).

### 3.5 Budesonide‐related side effects

No serious adverse side effects were observed. As previously published, 8 out of the 142 patients (6%) in our study group had shown initial growth retardation which was caught up by the 2 years follow‐up mark.^19^

## 4 DISCUSSION

Numerous studies have demonstrated a favorable impact of glucocorticoid administration on jaundice clearance at 6 months, leading to grade 1a evidence.^21^ However, out of several studies, only few were assessed for impact on NLS. Out of these, only five groups have reported on a beneficial effect of steroid administration on NLS (Table SZA). Therefore, the evidence justifying glucocorticoid administration to decrease or delay liver transplantation remains weak.

In a prospective study, Tyraskis et al. demonstrated that high‐dose systemic glucocorticoids may have a positive impact on NLS in patients younger than 45 days at surgery. Nevertheless, this hypothesis cannot be cannot reliably validated due the lack of a comparable control group.^21^ In contrast, Bezerra et al. found no evidence to support glucocorticoid use to improve NLS while observing some steroid associated events in their prospective randomized controlled trial (START trial).^17^ Davenport et al. demonstrated no benefit after the use of low‐dose glucocorticoids in a 2‐center randomized controlled trial.^22^ 2013 a study on high dose glucocorticoids was released by Davenport et al. showing a definite increase in clearance of jaundice with no improvement of NLS (log rank *p* = 0.51, longest follow‐up 5 years).^23^ Only one randomized controlled study by Lu et al. revealed significantly improved NLS at 24 months after i.v. and oral methylprednisolone therapy.^24^ Longest follow‐up in that study was 2 years.

Early jaundice clearance is considered indicative of favorable outcomes in the long run.^13^ We hypothesized that if glucocorticoid administration leads to improved clearance of jaundice, it would also positively impact NLS. However, considering the current data mostly suggesting improved jaundice clearance without NLS improvement following glucocorticoid administration,^23^, ^25^, ^26^ the hypothesis that glucocorticoid administration would positively impact NLS cannot be upheld without doubt and warrants further assessment.

Based on our own investigations, our department has ceased all administration of oral or intravenous glucocorticoids in 2008 after finding no beneficial effects.^27^

In 2021 on the other hand, a study group of our center reported promising results (improved NLS after budesonide application at 2‐years post‐Kasai) with the application of rectal budesonide administration, without serious adverse effects.^19^

Our current data suggests a sustained positive effect of rectal budesonide on NLS: Since our previous publication in 2021,^19^ 71 more patients in our patient cohort have completed the 2‐year follow‐up. The study group now also demonstrating improved outcomes for jfNLS at 6 months, with sustained beneficial results for jfNLS at 2 years, as well as overall NLS. Furthermore, this study indicates that this effect does persist over the years, as the results of our study group continue to outperform the control group at 5‐ and 10‐year follow‐up.

However, this effect is only observed in patients with non‐syndromic BA. Patients with syndromic BA did not appear to benefit from rectal budesonide. Syndromic forms are believed to develop during early pregnancy,^28^ whereas non‐syndromic BA appears to develop shortly after birth, driven by an inflammatory process.^29^ Budesonide, with its strong anti‐inflammatory properties^30^ could target these processes. The different treatment responses to budesonide between the two groups, would further support the hypothesis that inflammation isn't the central pathophysiological aspect in nonsyndromic patients.^31^

The degree of fibrosis or presence of cirrhosis did not seem to affect the overall response rates. Due to research suggesting reduced efficacy of budesonide in patients with liver fibrosis (particularly in the context of autoimmune hepatitis),^32^ we had expected different results. Many of the studies limited their follow‐up to shorter periods, which could explain the unsatisfactory NLS outcomes after systemic glucocorticoid administration.^22^, ^23^, ^26^ The positive effects of rectal budesonide administration only became evident in the 2‐year follow‐up in our previous study.^19^ Early benefits at 6 months were only observed once additional follow‐ups were completed (increasing the number of patients from 95 to 135 with completed 6 months follow‐up).

High‐dose systemic glucocorticoid administration typically begins with 4–5 mg/kg/day prednisolone or methylprednisolone.^11^, ^21^, ^26^ One of the most obvious advantages of systematic therapy being the possibility of precise weight‐adapted dosing. In contrast, rectal administration does not allow for such precision. A single dose of budesonide foam contains about 2 mg of budesonide, equivalent to 25 mg of prednisolone or 20 mg of methylprednisolone (Table SZB). For our study group, this roughly translates to a dose variation between 4 and 11 mg/kg/day. In our series, no serious steroid associated adverse effects were recorded, possible due to the first pass after rectal administration. As most of the medication is absorbed at the liver, therefore causing less systemic side effects.^33^ Oral budesonide, with its even more favorable pharmacokinetic profile in terms of first‐pass metabolism, may therefore represent a promising avenue for future investigation.^34^

The well‐established use of antibiotic treatment, ursodeoxycholic acid (UDCA), and vitamin substitution in our department highlights the importance of a multimodal approach to managing BA. While these therapies are part of standard care, the role of corticosteroids, be it by systemic or rectal administration, adds another layer of complexity.

This doesn't even include the other vast possibilities that are being examined at the moment.

Ileal Bile Acid Transporter Inhibitors (like Odevixibat and Maralixibat) are being tested for their ability to reduce pruritus by improving bile acid metabolism. Trials for BA showed promising results, with more research required to confirm safety and efficacy.^35^, ^36^ A recent study comprised of the patients included in the Childhood Liver Disease Research Network (ChiLDReN) and the Prospective Database of Infants with Cholestasis (PROBE; NCT00061828) introduced 6‐month serum bile acids as a positive prognostic factor for patients with normalized 6‐month serum bilirubin levels following HPE. The authors demonstrated a significant reduction in the cumulative incidence of 10‐year liver transplant or death in patients with a 6‐month bile acid level <40 μmol/L (8.5% vs. 42.9%). However, while bile acids could be identified as a marker, their influence on progression of liver damage remains unclear, warranting further randomized controlled trials^37^:

Whilst the BOLD study on Odevixibat is currently underway (NCT04336722), the EMBARK study on the effects of Maralixibat was recently completed. (NCT04524390)^38^ Even though the results have not been published yet, a press release was issued stating that the study failed the primary and secondary endpoints.^39^

Some centers administer bile acid sequestrants such as cholestyramine or phenobarbital to increase bile flow, despite the lack of beneficial evidence.^40^

Intravenous Immunoglobulins are tested for their immunomodulatory effects: but, initial trials have not shown any clear benefits.^41^, ^42^

Obeticholic acid is under investigation for its role in bile acid metabolism and inflammation,^43^ while N‐acetylcysteine is being studied for hepatoprotective properties^44^ Other investigated therapies include GCSF,^45^ Autologous bone marrow mononuclear cell infusion,^46^ Rituximab (ChiCTR2000031738), Bezafibrate^47^ (jRCTs031210066), and probiotics.^48^ These medications in the early stages of research exhibiting varying levels of promise. A study on the effects of intraoperative injection of Mitomycin‐C did not display a significant benefit.^49^

This study has several limitations: The retrospective nature of our data analysis allows for variability in the follow‐up protocols, potential biases (historical control group, change of surgeons) and confounding factors cannot be entirely ruled out. Despite this limitation, this study represents an extensive published series on adjuvant therapy for BA, comprising of 260 patients, providing valuable data.

So far, glucocorticoids—regardless of the mode of administration—are the most extensively studied adjuvant therapies in the postoperative management of BA. Our current us study is among few to demonstrate a positive effect on long‐term NLS following glucocorticoid administration. We attribute this success to our unique approach: by applying rectal budesonide foam, higher concentrations are delivered directly to the desired site of action while keeping systemic side effects at bay.

## 5 CONCLUSION

Our data suggest that rectal budesonide improves short‐ and long‐term jaundice‐free NLS after HPE in nonsyndromic BA patients, with less side effects than systemic glucocorticoids. These promising findings support the potential administration of rectal budesonide as standard postoperative medication after HPE. Given the observational nature of our data, prospective, randomized controlled trials with large sample sizes and long‐term follow‐up are crucial to confirm these results and determine the benefits and safety of this approach.

## CONFLICT OF INTEREST STATEMENT

The authors declare no conflicts of interest.

## Supporting information

Table X. Liver fibrosis (ISHAK scoring system^(20)^) at surgery.

Table Y. Postoperative bilirubin levels. Data are presented as median and interquartile range. n.s. = not significant.

Table Z. postoperative native liver survival of the study group in relation to liver fibrosis (ISHAK scoring system ^(20)^).

Table ZA. Study overview investigating effects of steroids on outcome after Kasai hepatoportoenterostomy (HPE). COJ = Clearance of jaundice. n.s.= not significant. NLS = native liver survival.

Table ZB. Dose equivalency overview.
