# Supplementary material for: Rectal budesonide: A potential game changer after Kasai hepatoportoenterostomy
Source: J Pediatr Gastroenterol Nutr. 2025 Jul 2;81(3):626–33. doi: 10.1002/jpn3.70147 (PMC12408950; doi:10.1002/jpn3.70147)
Supplement: Supplementary file 1 — Table X. Liver fibrosis (ISHAK scoring system(20)) at surgery. [file JPN3-81-626-s002.docx]

|  | **Study group (n=142)** | **Control group (n=118)** | **p-Value** |
| --- | --- | --- | --- |
| ISHAK 1 | 2.1% (3) | 3.4% (4) | n.s. |
| ISHAK 2 | 16.2% (23) | 5.1% (6) |  |
| ISHAK 3 | 19% (27) | 15.3 (18) |  |
| ISHAK 4 | 26.1% (37) | 7.6% (9) |  |
| ISHAK 5 | 21.8% (31) | 7.6% (9) |  |
| ISHAK 6 | 9.2% (16) | 4.2% (5) |  |
| not available | 5.6% (8) | 56.8% (67) |  |

Table X: Liver fibrosis (ISHAK scoring system) at surgery
