# Supplementary material for: Rectal budesonide: A potential game changer after Kasai hepatoportoenterostomy
Source: J Pediatr Gastroenterol Nutr. 2025 Jul 2;81(3):626–33. doi: 10.1002/jpn3.70147 (PMC12408950; doi:10.1002/jpn3.70147)
Supplement: Supplementary file 2 — Table Y. Postoperative bilirubin levels. Data are presented as median and interquartile range. n.s. = not significant. [file JPN3-81-626-s004.docx]

| **Serum bilirubin (μmol/L)** | **Study group** | **Control group** | **p-Value** |
| --- | --- | --- | --- |
| 6 months | 9 (4-48) | 33 (9-211) | **<0.005** |
| 1 year | 5.5 (3-22) | 8 (4-38) | n.s. |
| 2 years | 5.5 (3-11) | 8 (1-16) | **<0.05** |
| 5 years | 6 (4-8) | 8 (5-11) | n.s. |
| 10 years | 8 (5-10) | 7 (4-10) | n.s. |

Table Y: Postoperative bilirubin levels
