# Supplementary material for: Rectal budesonide: A potential game changer after Kasai hepatoportoenterostomy
Source: J Pediatr Gastroenterol Nutr. 2025 Jul 2;81(3):626–33. doi: 10.1002/jpn3.70147 (PMC12408950; doi:10.1002/jpn3.70147)
Supplement: Supplementary file 3 — Table Z. postoperative native liver survival of the study group in relation to liver fibrosis (ISHAK scoring system (20)). [file JPN3-81-626-s001.docx]

|  | **Transplant/death** | **Native liver survival** | **p-value** |
| --- | --- | --- | --- |
| ISHAK 1 | 1.2% (1) | 3.3% (2) | n.s. |
| ISHAK 2 | 15.4% (12) | 18.3% (1)1 |  |
| ISHAK 3 | 17.9% (14) | 20% (12) |  |
| ISHAK 4 | 23.1% (18) | 28.3% (17) |  |
| ISHAK 5 | 22% (17) | 21.7% (13) |  |
| ISHAK 6 | 12.8% (10) | 5% (3) |  |
| Not available | 7.7% (6) | 3% (2) |  |

*Table 7: Postoperative native liver survival of the study group in relation to liver fibrosis (ISHAK score)*
