# Supplementary material for: Rectal budesonide: A potential game changer after Kasai hepatoportoenterostomy
Source: J Pediatr Gastroenterol Nutr. 2025 Jul 2;81(3):626–33. doi: 10.1002/jpn3.70147 (PMC12408950; doi:10.1002/jpn3.70147)
Supplement: Supplementary file 4 — Table ZA. Study overview investigating effects of steroids on outcome after Kasai hepatoportoenterostomy (HPE). COJ = Clearance of jaundice. n.s.= not significant. NLS = native liver survival. [file JPN3-81-626-s005.docx]

| **Lead author** | **Glucocorticoid** | **Study design** | **Sample size** | **Results** |
| --- | --- | --- | --- | --- |
| Davenport 2007^(1)^ | Prednisolone 2mg/kg/d vs. none | RCT | 71 | Clearance of jaundice (COJ) n.s.  NLS n.s. |
| Davenport 2013^(2)^ | Prednisolone 5mg/kg/d vs. 2mg/kg/d vs. none | Prospective, open-label study | 152 | COJ significant improvement  NLS n.s. |
| Bezerra 2014^(3)^ | Methylprednisolone (Dose-equivalent Prednisolone 5mg/kg/d) vs. none | RCT | 140 | 6 months Bilirubin n.s.  NLS n.s. |
| Muraji Higashimoto 2007 | Prednisolone  High dose (>4mg/kg/d) vs. Low dose (<4mg/kg/d) vs. none | Cohort | 222 | NLS : significant improval when comparing only high dose steroid group vs. no steroid group (p<0.05) |
| Japanese Biliary Atresia Society (2013)^(4)^ | Prednisolone  High dose (4mg/kg/d) vs. Low dose (2mg/kg/d) | Multicentre RCT | 69 | Bilirubin levels significantly lower in high dose group if patients <70 days  NLS not investigated |
| Petersen 2008^(5)^ | Methylprednisolone (Dose-equivalent Prednisolone 12.5mg/kg/d starting dose) vs. none | Prospective, open label | 49 | NLS at 6 months & 2 years: n.s. |
| Lu 2023^(6)^ | Methylprednisolone (Dose-equivalent Prednisolone 5mg starting dose) vs. none | RCT | 200 | NLS and CoJ 6,12 & 24 months: significant improval in steroid group  Kaplan Meier analysis: significant longer NLS (p=0.02) |
| Meyers 2003^(7)^ | Methylprednisolone (Dose-equivalent Prednisolone 12.5mg/kg/d starting dose) vs. none | Cohort | 28 | NLS 5 years: significant improval in steroid group  (79% vs 15%) |
| Kobayashi 2005^(8)^ | Prednisolone (4 dosage groups, up to 20mg/kg/d starting dose) vs. none | Prospective, open-label study | 63 | CoJ and “time to CoJ” significantly improved for high dosage, multiple cycles group (vs. no glucocorticoids or any of the other single cycle dosage groups) |
| Escobar 2006^(9)^ | Varied, Prednisone and dexamethasone between 6mg/kg/d-20mg/kg/d starting dose | Cohort | 43 | CoJ 6 months: significant improval in steroid group (p=0.01)  NLS: n.s. |
| Vejchapipat 2007^(10)^ | Prednisolone 4 mg/kg/d vs. none | Cohort | 53 | COJ 6 months: n.s. |
| Chung 2008^(11)^ | Prednisolone 4 mg/kg/d vs. none | Cohort | 30 | NLS (longest Follow-up 15 months): n.s.  CoJ: n.s.  Mean Bilirubin at 3 & 6 months significantly improved (p<0.01) |
| Dong 2013^(12)^ | Prednisone (Dose-equivalent Prednisolone 4 mg/kg/d starting dose) intravenous vs oral | Cohort | 380 | NLS 2 years: significant improvement in “high dose” iv group vs. “low dose” oral group (53.3% vs. 38.3%, p<0.01)  CoJ at 3, 6, 12 months significantly improved (p<0.01) |
| Kuebler 2021^(13)^ | Budesonide rectal (Dose-equivalent Prednisolone 4.5-10mg/kg/d) vs. none | Cohort | 176 | NLS 6 months: n.s.  NLS 2 years: significant improvement in steroid group (54% vs 32%, p<0.001) |

Table ZA: Study overview investigating effects of steroids on outcome after HPE

1. Davenport M, Stringer MD, Tizzard SA, et al. Randomized, double‐blind, placebo‐controlled trial of corticosteroids after Kasai portoenterostomy for biliary atresia. *Hepatology* 2007; 46:1821–1827. <https://doi.org/10.1002/hep.21873>

2. Davenport M, Parsons C, Tizzard S, et al. Steroids in biliary atresia: Single surgeon, single centre, prospective study. *J Hepatol* 2013; 59:1054–1058. <https://doi.org/10.1016/j.jhep.2013.06.012>

3. Bezerra JA, Spino C, Magee JC, et al. Use of Corticosteroids After Hepatoportoenterostomy for Bile Drainage in Infants With Biliary Atresia: The START Randomized Clinical Trial. *JAMA* 2014; 311:1750–1759. <https://doi.org/10.1001/jama.2014.2623>

4. Society JBA, Nio M, Muraji T. Multicenter randomized trial of postoperative corticosteroid therapy for biliary atresia. *Pediatr Surg Int* 2013; 29:1091–1095. <https://doi.org/10.1007/s00383-013-3377-6>

5. Petersen C, Harder D, Melter M, et al. Postoperative High-Dose Steroids Do Not Improve Mid-Term Survival With Native Liver in Biliary Atresia. *Am J Gastroenterol* 2008; 103:712–719. <https://doi.org/10.1111/j.1572-0241.2007.01721.x>

6. Lu X, Jiang J, Shen Z, et al. Effect of Adjuvant Steroid Therapy in Type 3 Biliary Atresia. *Ann Surg* 2023; 277:e1200–e1207. <https://doi.org/10.1097/sla.0000000000005407>

7. Meyers RL, Book LS, O’Gorman MA, et al. High-dose steroids, ursodeoxycholic acid, and chronic intravenous antibiotics improve bile flow after Kasai procedure in infants with biliary atresia. *J Pediatr Surg* 2003; 38:406–411. <https://doi.org/10.1053/jpsu.2003.50069>

8. Kobayashi H, Yamataka A, Koga H, et al. Optimum prednisolone usage in patients with biliary atresia postportoenterostomy. *J Pediatr Surg* 2005; 40:327–330. <https://doi.org/10.1016/j.jpedsurg.2004.10.017>

9. Escobar MA, Jay CL, Brooks RM, et al. Effect of corticosteroid therapy on outcomes in biliary atresia after Kasai portoenterostomy. *J Pediatr Surg* 2006; 41:99–103. <https://doi.org/10.1016/j.jpedsurg.2005.10.072>

10. Vejchapipat P, Passakonnirin R, Sookpotarom P, et al. High-dose steroids do not improve early outcome in biliary atresia. *J Pediatr Surg* 2007; 42:2102–2105. <https://doi.org/10.1016/j.jpedsurg.2007.08.038>

11. Chung HY, Wong KKY, Lan LCL, et al. Evaluation of a standardized protocol in the use of steroids after Kasai operation. *Pediatr Surg Int* 2008; 24:1001–1004. <https://doi.org/10.1007/s00383-008-2200-2>

12. Dong R, Song Z, Chen G, et al. Improved Outcome of Biliary Atresia with Postoperative High‐Dose Steroid. *Gastroenterol Res Pr* 2013; 2013:902431. <https://doi.org/10.1155/2013/902431>

13. Kuebler JF, Madadi-Sanjani O, Pfister ED, et al. Adjuvant Therapy with Budesonide Post-Kasai Reduces the Need for Liver Transplantation in Biliary Atresia. *J Clin Med* 2021; 10:5758. <https://doi.org/10.3390/jcm10245758>
