# Supplementary material for: Rectal budesonide: A potential game changer after Kasai hepatoportoenterostomy
Source: J Pediatr Gastroenterol Nutr. 2025 Jul 2;81(3):626–33. doi: 10.1002/jpn3.70147 (PMC12408950; doi:10.1002/jpn3.70147)
Supplement: Supplementary file 5 — Table ZB. Dose equivalency overview. [file JPN3-81-626-s003.docx]

| **Glucocorticoid** | **Equivalent Dose** |
| --- | --- |
| Cortisone | 25 mg |
| Hydrocortisone | 20 mg |
| Prednisone | 5 mg |
| Prednisolone | 5 mg |
| Methylprednisolone | 4 mg |
| Betamethasone | 0.6 – 0.75 mg |
| Dexamethasone | 0.75 mg^(1)^ |
| Budesonide | 0,4 mg ^(2)^ |

Table ZB: Dose equivalency overview

1. Samuel S, Nguyen T, Choi HA. Pharmacologic Characteristics of Corticosteroids. *J Neurocritical Care* 2017; 10:53–59. <https://doi.org/10.18700/jnc.170035>

2. Rutgeerts P, Lofberg R, Malchow H, et al. A Comparison of Budesonide with Prednisolone for Active Crohn’s Disease. *N Engl J Med* 1994; 331:842–845. <https://doi.org/10.1056/nejm199409293311304>
